# Performance evaluation of *Moringa oleifera* seeds aqueous extract for removing *Microcystis aeruginosa* and microcystins from municipal treated-water

**DOI:** 10.3389/fbioe.2023.1329431

**Published:** 2024-02-01

**Authors:** Haifa A. S. Alhaithloul, Zakaria A. Mohamed, Abdullah A. Saber, Ibtisam Mohammed Alsudays, Mohamed A. Abdein, Mesfer M. Alqahtani, Noha G. AbuSetta, Amr Elkelish, Leonardo Martín Pérez, Fauzeya Mateq Albalwe, Asmaa A. Bakr

**Affiliations:** ^1^ Department of Biology, College of Science, Jouf University, Sakaka, Saudi Arabia; ^2^ Microbiology and Botany Department, Faculty of Science, Sohag University, Sohag, Egypt; ^3^ Botany Department, Faculty of Science, Ain Shams University, Cairo, Egypt; ^4^ Department of Biology, College of Science and Arts, Qassim University, Unaizah, Saudi Arabia; ^5^ Seeds Development Department, El-Nada Misr Scientific Research and Development Projects, Mansoura, Egypt; ^6^ Department of Biological Sciences, Faculty of Science and Humanities, Shaqra University, Shaqraa, Saudi Arabia; ^7^ Microbiology and Botany Department, Faculty of Science, South Valley University, Qena, Egypt; ^8^ Department of Biology, College of Science, Imam Muhammad bin Saud Islamic University (IMSIU), Riyadh, Saudi Arabia; ^9^ Department of Botany and Microbiology, Faculty of Science, Suez Canal University, Ismailia, Egypt; ^10^ Facultad de Química e Ingeniería del Rosario, Pontificia Universidad Católica Argentina (UCA), Rosario, Argentina; ^11^ Laboratory of Environmental and Sanitary Microbiology (MSMLab-UPC), Universitat Politècnica de Catalunya-BarcelonaTech, Terrassa, Spain; ^12^ Department of Biology, Faculty of Science, University of Tabuk, Tabuk, Saudi Arabia

**Keywords:** cyanotoxins, *Microcystis aeruginosa*, *Moringa oleifera*, natural coagulant, water treatment, HPLC-DAD

## Abstract

**Introduction:** Toxic microcystins (MCs) produced by cyanoprokaryotes -particularly by the cosmopolitan cyanobacterium *Microcystis aeruginosa*- pose adverse effects on aquatic organisms and their ecosystem and may also cause serious impacts on human health. These harmful monocyclic heptapeptides are the most prevalent cyanotoxins reported in freshwaters and must be eliminated for avoiding MCs release in receiving water bodies. Hence, this work aimed to test the efficacy of *Moringa oleifera* seeds water-based extract (MO) as a natural coagulant for removing cyanobacteria (especially *M. aeruginosa*), microalgae, and its associated MCs from pre-treated municipal wastewaters.

**Methodology:** Four different MO coagulant doses (25, 50, 75 and 100 mg L^−1^) were investigated for cyanobacteria and microalgae removal by conventional coagulation assays and morphology-based taxonomy studies. Additionally, water turbidity and chlorophyll *a* (Chl *a*) content were also determined. Further, the presence and concentration of MCs soluble in water, remaining in the particulate fraction, and flocculated within the residual sludge were assessed using high-performance liquid chromatography coupled with diode array detection (HPLC-DAD).

**Results:** The treatment with MO at 100 mg L^−1^ substantially reduced the number of cyanobacterial and microalgal species in the treated samples (average removal rate of 93.8% and 86.9%, respectively). These results agreed with a ∼44% concomitant reduction in Chl a and ∼97% reduction in water turbidity (a surrogate marker for suspended solids content). Notably, MCs concentrations in the treated water were significantly lowered to 0.6 ± 0.1 µg L^−1^ after addition of 100 mg L^−1^ MO. This value is below the WHO recommended limits for MCs presence in drinking water (<1.0 µg L^−1^).

**Discussion:** The present study provides promising insights into the applicability of MO as a cost-effective, reliable, and sustainable natural coagulant, particularly for using in developing countries, to eliminate harmful cyanobacteria and cyanotoxins in municipal water treatment facilities.

## 1 Introduction

Water is undoubtedly the most vital element among the natural resources. In many developing and low-income countries, the access to clean and safe water is a crucial issue. Every year, hundreds of people die because of water-borne diseases. Susceptible countries pay high costs to import chemicals for water treatment. Therefore, voices have raised to develop cost-effective, safe, and more sustainable processes for drinking water obtention.

With the increasing regional temperatures and the ongoing climate crisis, cyanobacterial blooms in natural water bodies are more frequent. Its intensive growth not only affects the surrounding ecosystems (e.g., water eutrophication, dissolve oxygen depletion, hazardous compounds release, etc.), but also impacts on the aquatic biota, and even on public health. As a consequence, recreational activities (e.g., boating, diving, and fishing), aquaculture practices, and also drinking water production are restricted (Merel et al., 2013; Chapman, 2015; He et al., 2016).

Cyanotoxins are secondary metabolites produced by toxic cyanobacteria. These water-soluble compounds are classified as neurotoxins (e.g., saxitoxin, homoanatoxin-a, and anatoxin-a) or hepatotoxins (e.g., nodularin, cylindrospermopsin, and microcystins) based on their chemical structure and the cell/tissue types they affect (Kaebernick and Neilan, 2001; Corbel et al., 2014; Rastogi et al., 2014). The genera *Microcystis*, *Planktothrix*, *Anabaena*, *Oscillatoria*, and *Nostoc* are the mainly producers of the most common and prevailing microcystins (MCs) (Sivonen and Hudnell, 2008; Lopes and Vasconcelos, 2011). These compounds are biosynthesized non-ribosomally via microcystin synthetase gene clusters (*mcy*) consisting of polyketide synthases, non-ribosomal peptide synthetases, and tailoring enzymes (Tillett et al., 2000). More importantly, MCs are heat-stable and highly toxic peptides (Krishnan and Mou, 2021), therefore their efficient removal in water treatment facilities is of paramount importance. Accordingly, coagulation processes can be applied for MCs removal from water (Gouvea-Barros et al., 2019). As such, the application of natural coagulants is preferred since they are safe, non-toxic, cost-effective, and environmentally friendly (Koul et al., 2022). These compounds are frequently manufactured from plant seeds, leaves, and roots; and have been used for thousands of years in countries like India, Africa, and China (Dhakad et al., 2019; Yadav and Meena, 2021). Natural coagulants offer a better option because they are not only safe for human health, but also less expensive than conventional chemicals regarding their local availability in most rural communities. In addition, they have a low-cost of production and a high biodegradability (Koul et al., 2022; Yadav and Meena, 2021; Bolto and Gregory, 2007).

Recent studies have emphasized the coagulative properties of *M*. *oleifera* seeds (Gouvea-Barros et al., 2019; Okuda et al., 2001a; Okuda et al., 2001b). In Middle Eastern countries, *Moringa* seed powder has been found to be a suitable option for several purposes, including water treatment, mostly in poor regions where sanitary solutions are scarce (Gopalakrishnan et al., 2016; Rani and Arumugam, 2017; Dhakad et al., 2019). When mixed with water, *Moringa* seeds yield positively charged water-soluble proteins that attract the negative charge colloidal particles (e.g., cyanobacterial and algal cells, organic matter, polar contaminants, etc.), promoting floc formation and agglomeration (Yap et al., 2014; Sun et al., 2020; Ghernaout et al., 2020; Nhut et al., 2021; Abdul Hamid et al., 2016). In addition, cyanobacterial biomass removal without affecting cell integrity to avoid intracellular toxins release is also highly desirable. However, there is still very limited information about the use of *M. oleifera* extract*s* for *M*. *aeruginosa* and MCs removal from municipal untreated waters (Gouvea-Barros et al., 2019). Hence, in this work we evaluated the performance of *M. oleifera* seeds aqueous extract (MO) for removing *M. aeruginosa* population as well as MCs from water samples provided by a local water treatment facility. The promising results here obtained could be further used for the development of efficient and cost-effective eco-biotechnological approaches for safe water production, particularly in climate-susceptible developing and low-income countries.

## 2 Materials and methods

### 2.1 Preparation of *M. oleifera* seeds aqueous extract

*M. oleifera* seeds (250 g) were obtained from a local market (Cairo, Egypt) and air-dried for 24 h. Afterward, the pre-dried seeds were powderized using a kitchen blender. Then, 1.0 g of seed powder was suspended in 100 mL distilled water (ca. 10,000 mg L^−1^), boiled for 30 min, and vacuum-filtered through Whatman No. 5 paper (Fisher Scientific, Pittsburgh, PA) after the solution cooled down at room temperature. The *M. oleifera* seeds aqueous extract (MO) was prepared and immediately used in the coagulation studies without prior delay.

### 2.2 Coagulation studies

Water samples for testing were collected from the drinking water and sanitation treatment plant located in Akhmim (Sohag, Egypt), and transported to the laboratory under ambient conditions. Coagulation studies were performed in a conventional jar-test apparatus at 25.0°C ± 3.0°C. Samples (200 mL) were transferred to the jar-test and different coagulant dosage (0, 25, 50, 75, and 100 mg L^−1^ MO) were supplied. The experiments were carried out without prior pH adjustment (pH∼6.7–6.9) in a three-steps process: fast mixing at 95 rpm for 1 min, followed by a slow mixing period for 15 min at 10 rpm, and finally settled for 1 h. After settling, the supernatant was withdrawn from the beaker and transferred to a flask for subsequent analyses (cyanobacterial and microalgal count, turbidity, chlorophyll *a* content determination, and MCs quantification). Additionally, the sludge (*i.e.*, dense floc) was stored for cyanobactrial count and MCs quantification. Additionally, water turbidity was measured in triplicate (*n* = 3) using a HACH DR/2010 spectrophotometer (Hach Company, Loveland, United States) following the procedures described by the APHA standard methods (APHA, 1995) and reported as mg L^−1^ ± standard deviation (S.D.) of suspended solids (3 NTU = 1 mg L^−1^).

### 2.3 Cyanobacteria and algae identification

Cyanobacterial and algal taxa present in the coagulant-treated water samples and the corresponding flocculated sludge were morphologically identified following different taxonomical keys described by (Lichtfouse et al., 2019; Yamaguchi et al., 2021; El Bouaidi et al., 2022). A Sedgewick-Rafter chamber was used for counting the number of taxonomic dissimilar units (i.e., individual cells or colonies) per mL of sample in a Zeiss Axiovert 40 CFL microscope (Carl Zeiss, Oberkochen, Germany). Briefly, 1 mL of control or MO-treated water sample was placed onto the chamber and a special coverslip was laid across the top of the chamber, assuring that no bubbles were trapped beneath the coverslip. The chamber was left for about half-an-hour for cells sedimentation. The cyanobacterial and microalgal taxa were then counted from one corner of the counting cell to the other. In the case of colony of cyanobacteria (e.g., *M. aeruginosa*) one colony was considered as one organism. In the case of microalgae that are distinguished as individual cells or filaments (e.g., *Euglena* sp., *Chlorella* sp., *Cylindrospermopsis* sp.) each cell was considered as an individual. The cyanobacteria and microalgae concentrations were calculated as stated in the standard methods for the examination of water and wastewater (APHA, 1995). Results were expressed as individuals/organisms mL^−1^ ± standard deviation (S.D.) from an average of three replicates (*n* = 3). Further, the removal efficiency (%) for each species was calculated based on the number of individuals/organisms counted as follows:

$$\text{Removal efficiency }\left(\%\right)=\left[\left(C_{1}-C_{2}\right)/C_{1}\right]\times 100$$

where, C_1_ and C_2_ are the number of different taxonomic units before (i.e., control) and after MO treatment, respectively. Finally, the overall removal (%) for each MO treatment was calculated as the mean value of the different removal efficiency (%) obtained for each species.

### 2.4 Determination of chlorophyll a

Chlorophyll *a* (Chl *a*) was determined by methanolic extraction and quantified using a HACH DR/2010 spectrophotometer (Hach Company, Loveland, 104 United States of America). Briefly, 10.0 mL water sample was filtered through a Whitman GF/C glass fiber filter. Afterwards, 10.0 mL of 80% methanol (Merck, Darmstadt, Germany) were added to the filter and incubated in the dark at 4°C for 24 h. Finally, the absorbance of the supernatant solution was measured at 663 and 644 nm. The contents of Chl *a* was estimated according to the equation described by (Carralero Bon et al., 2021) and expressed as μg mL^−1^ sample. Data were reported as the mean value ± standard deviation (S.D.) of three replicates (*n* = 3).

### 2.5 Microcystins (MCs) assessment

MCs concentration were assessed following the protocol described by (Yasmin et al., 2019). Briefly, 10.0 mL of coagulant-treated water sample was filtered using a Whitman GF/C glass fiber filter. The supernatant was stored and processed for the quantification of soluble MCs (*i.e.*, “dissolved MCs”). Afterwards, 20 mL of 80% methanol (Merck, Darmstadt, Germany) was added to the filter and incubated in the dark at room temperature for 24 h to extract the MCs present in the particulate fraction (“particulate MCs”). Additionally, sludge samples (∼50 mg) were similarly treated with 80% methanol (Merck, Darmstadt, Germany) and dark-incubated (24 h, room temperature) to extract the remanent MCs in the sludge fraction (“MCs sludge”). All methanolic solutions were stored at 4°C in glass vessels. The MC-LR standard was purchased from ENZO Life Science (England. United Kingdom). All other reagents used were HPLC grade. A stock solution (10.0 mg L^−1^) was prepared by dissolving 0.100 mg of MC-LR standard in 10.00 mL methanol. Later, MC-LR standard solutions were prepared at concentrations of 0, 20, 40, 80 and 100 μg L^−1^ by adding the corresponding volume of stock solution to methanol. High-performance liquid chromatography (HPLC) was used to detect and quantify the MCs concentration in the three different fractions (*i.e.*, “dissolved MCs”, “particulate MCs”, and “MCs sludge”) (Supplementary Figure S1). An Agilent Technologies 1200 Series HPLC system (Agilent Technologies, Santa Clara, CA, United States) equipped with a photodiode array detector (DAD) and outfitted with a Zorbax Eclipse XDB-C18 reversed-phase column (150 mm × 4.6 mm, particle size 5 μm; Agilent Technologies) was used for the separation. An optimal elution solution composed by 100% (*v*/*v*) methanol and 0.05% *v*/*v* trifluoroacetic acid (60:40 *v*/*v*) was applied over 30 min at a flow rate of 1 mL min^−1^, while the column temperature was kept at 30°C. The UV wavelength of the DAD detector was set from 200 to 300 nm. All calibration processes displayed reproducible linear relationships (R^2^ > 0.98).

### 2.6 Statistical analysis

The results were statistically analyzed using the SPSS program version 22.0 (IBM SPSS Statistics for Windows, Armonk, NY: IBMCorp, 2013). The ANOVA test was used to compare data between groups. When the differences in the measured values were significant different the Tukey’s honest *post hoc* test was applied at a confident interval of 95% (*p* < 0.05).

## 3 Results

**FIGURE 1:**
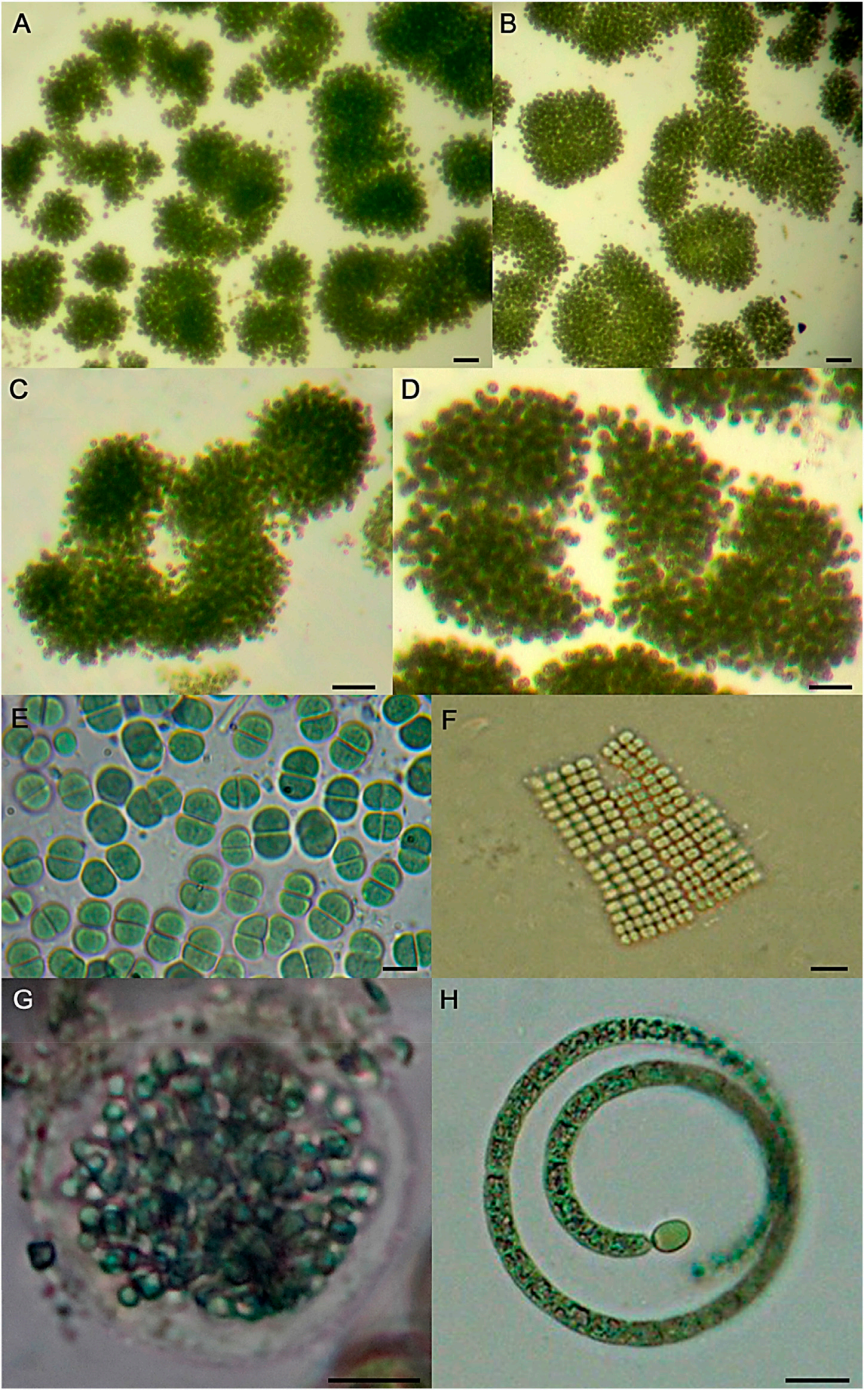
Representative light micrographs of cyanobacterial species recorded in the present study. **(A–D)** different colonies of *Microcystis aeruginosa,* **(E)** *Chroococcus* sp., **(F)** *Merismopedia* sp., **(G)** *Aphanocapsa* sp., **(H)** *Cylindrospermopsis* sp. Scale bars: 40 µm **(A, B)**, 20 µm **(C, D)**, 10 µm **(E–H)**.

**FIGURE 2:**
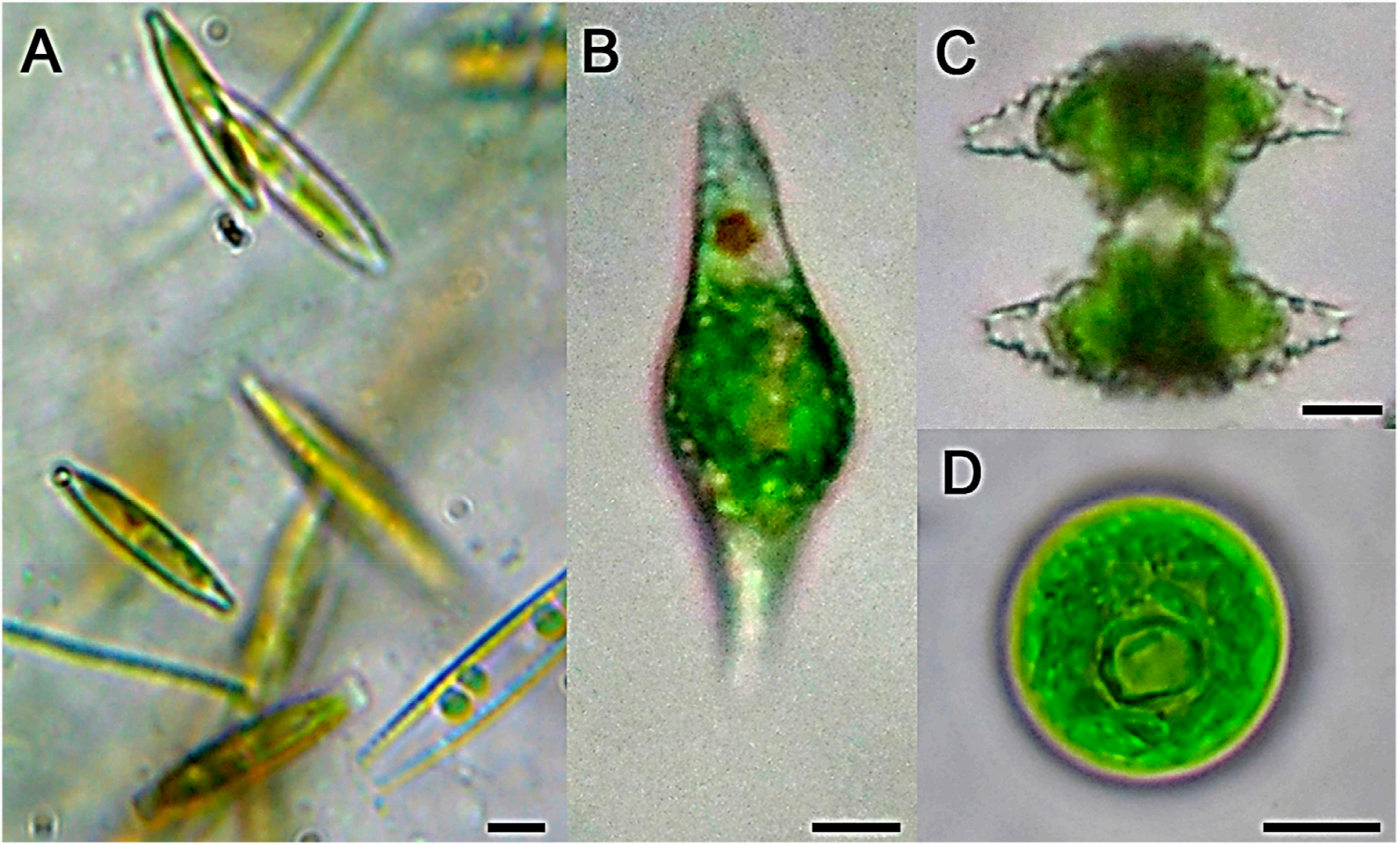
Representative light micrographs of some microalgal species recorded in the present study. **(A)** *Nitzschia* sp., **(B)** *Euglena* sp., **(C)** *Staurastrum* sp., **(D)** *Chlorococcum* sp. Scale bars: 10 µm.

A total of eleven species of cyanobacteria and other microalgae groups were identified and classified following taxonomic keys and descriptions (Figures 1, 2). Members of the phylum Cyanobacteria including *M. aeruginosa*, *Merismopedia* sp., *Aphanocapsa* sp., *Chroococcus* sp., and *Cylindrospermopsis* sp. dominated the tested waters (∼95.5% of the total taxonomic unit count), while other microalgal taxa were less represented (Table 1).

**TABLE 1:** Abundance of cyanobacterial and microalgal taxa (individuals/organisms mL^−1^) recorded in the municipal treated-water samples after addition of different concentrations of *M. oleifera* seeds aqueous extract (MO). Removal efficiency (%) for each species is indicated into brackets. The overall removal (%) was calculated as the mean value of the removal efficiencies (%) for each species.

Taxa	MO (mg L^−1^)				
Cyanobacteria	0 (control)	25	50	75	100
*Microcystis aeruginosa*	476 ± 5^e^	224 ± 3^d^ (52.9%)	196 ± 3^c^ (58.8%)	98 ± 6^b^ (79.4%)	56 ± 1^a^ (88.2%)
*Aphanocapsa* sp	434 ± 3^e^	252 ± 2^d^ (41.9%)	126 ± 5^c^ (71.0%)	70 ± 4^b^ (83.9%)	56 ± 5^a^ (87.1%)
*Merismopedia* sp	192 ± 2^e^	96 ± 2^d^ (50.0%)	64 ± 3^c^ (66.7%)	32 ± 2^b^ (83.3%)	0 ± 0^a^ (100%)
*Cylindrospermopsis* sp	100 ± 4^b^	0 ± 0^a^ (100%)	0 ± 0^a^ (100%)	0 ± 0^a^ (100%)	0 ± 0^a^ (100%)
*Chroococcus* sp	62 ± 2^e^	26 ± 1^d^ (58.1%)	10 ± 2^c^ (83.9%)	6 ± 2^b^ (90.3%)	4 ± 2^a^ (93.5%)
Overall removal (%)	0	60.6	76.1	87.4	93.8

Microalgae	0 (control)	25	50	75	100
*Nitzschia* sp	8 ± 3^a^	7 ± 4^a^ (12.5%)	4 ± 3^a^ (50.0%)	3 ± 2^a^ (62.5%)	3 ± 2^a^ (62.5%)
*Euglena* sp	6 ± 3^b^	3 ± 2^ab^ (50.0%)	1 ± 1^a^ (83.3%)	0 ± 0^a^ (100%)	0 ± 0^a^ (100%)
*Chlorella* sp	19 ± 7^b^	8 ± 6^a^ (57.9%)	7 ± 2^a^ (63.2%)	4 ± 3^a^ (78.9%)	2 ± 1^a^ (89.5%)
*Staurastrum* sp	10 ± 2^b^	7 ± 6^ab^ (30.0%)	5 ± 2^ab^ (50.0%)	4 ± 2^ab^ (60.0%)	3 ± 1^a^ (70.0%)
*Chlorococcum* sp	4 ± 3^b^	2 ± 2^ab^ (50%)	0 ± 0^a^ (100%)	0 ± 0^a^ (100%)	0 ± 0^a^ (100%)
*Crucigenia* sp	13 ± 2^b^	0 ± 0^a^ (100%)	0 ± 0^a^ (100%)	0 ± 0^a^ (100%)	0 ± 0^a^ (100%)
Overall removal (%)	0	50.1	74.4	83.6	86.9

Data are mean ± S.D. of three replicates (*n* = 3). Different lowercase letters in the same row represent significant statistical difference (*p* < 0.05) (e.g., “a” and “b” are statistically different from each other, but not from “ab”).

All four MO dosage substantially reduced the numbers of cyanobacteria and other microalgae in the treated samples. In particular, 100 mg L^−1^ of the natural coagulant exhibited the highest performance in reducing the number of total cells (93.8% cyanobacteria and 86.9% microalgae reduction compared to the control group) (Table 1). Moreover, *Cylindrospermopsis* sp. was completely eliminated from water samples treated with all four MO concentration (25, 50, 75 and 100 mg L^−1^), while *Merismopedia* sp. was only eliminated at the highest MO concentration tested (100 mg L^−1^).

**FIGURE 3:**
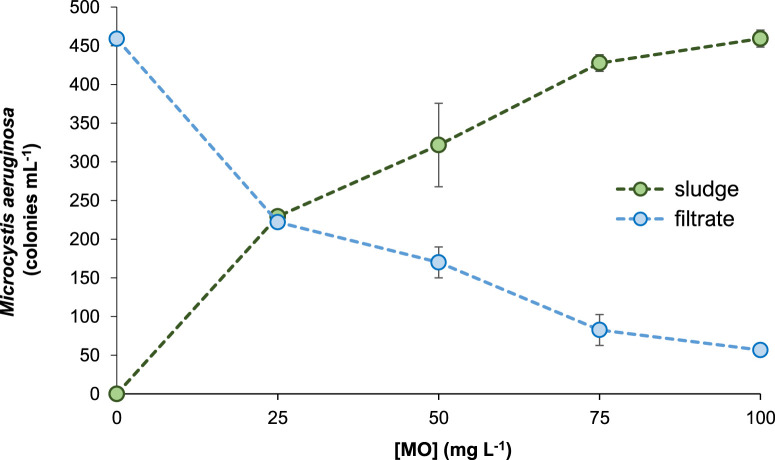
Abundance of *Microcystis aeruginosa* in the supernatant (filtrate) and the floc phase (sludge) of municipal treated-water after coagulation with different concentrations of *Moringa oleifera* seeds aqueous extract (MO). Data are mean ± S.D. of three independent experiments (*n* = 3).

The observed reduction in the amount of cyanobacteria and microalgae in suspension agreed with an increase of algae presence in the sludge (Figure 3).

**FIGURE 4:**
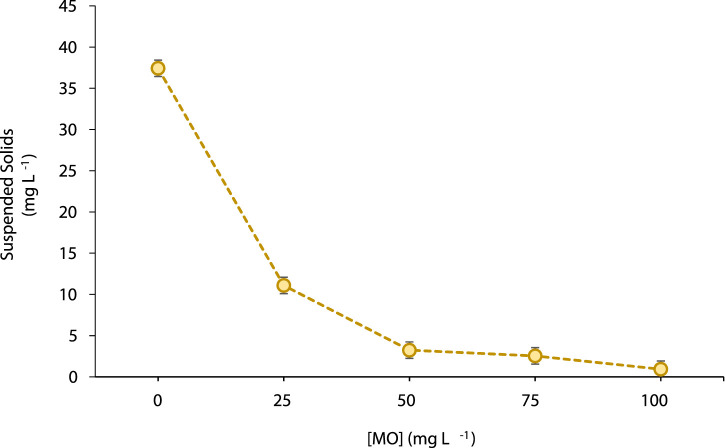
Suspended solids (mg L^−1^) concentration in the supernatant of municipal treated-water after coagulation with different concentrations of *Moringa oleifera* seeds aqueous extract (MO). Data are mean ± S.D. of three independent experiments (*n* = 3).

The control sample exhibited the highest Chl *a* concentration (176.3 μg mL^−1^) as well as the highest content of suspended solids (Table 2; Figure 4). When increasing MO dosage in the range 25–100 mg L^−1^ the concentration of Chl *a* gradually decreased. The lower Chl *a* content (99.3 μg ml^−1^) was reported for samples treated with 100 mg L^−1^ of coagulant (Table 2).

**TABLE 2:** Chlorophyll *a* (Chl *a*) concentration in the supernatant of municipal treated-water after coagulation with different concentrations of *Moringa oleifera* seeds aqueous extract (MO).

MO (mg L^−1^)	Chl *a* concentration (µg mL^−1^)
0 (control)	176.3 ± 14.8^d^
25	171.7 ± 7.6^d^
50	151.6 ± 6.1^c^
75	125.1 ± 5.1^b^
100	99.3 ± 4.0^a^

Data are mean ± S.D. of three replicates (*n* = 3). Different lowercase letters represent significant statistical difference (*p* < 0.05).

A similar behaviour was observed for the amount of solids in suspension. In this case, the values significantly decreased (*p* < 0.05) from 36.8 mg L^−1^ in the MO-untreated control samples to 0.2 mg L^−1^ when using 100 mg L^−1^ of MO. Notably, more than 90% of suspended solids removal was achieved for coagulant doses ≥50 mg L^−1^ (Figure 4).

**FIGURE 5:**
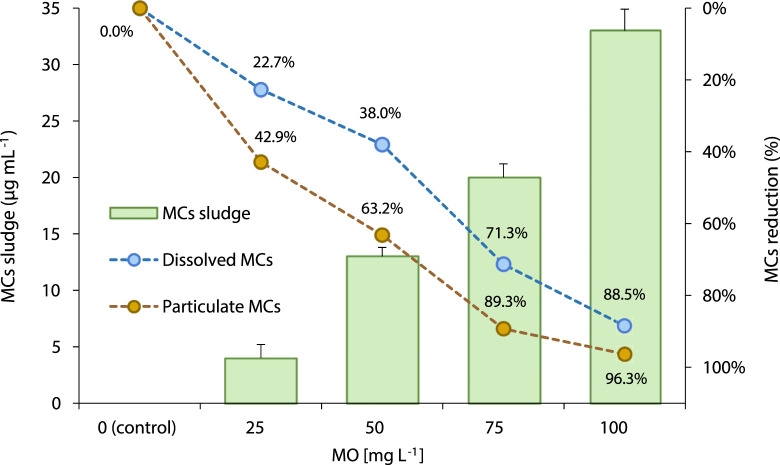
Distribution (%) of MCs soluble in water (dissolved MCs), remaining in the particulate fraction (particulate MCs), and flocculated within the residual sludge (MCs sludge) after *Moringa oleifera* seeds aqueous extract (MO) addition on municipal treated-water samples.

MO efficiently removed toxic MCs from water samples (Table 3; Figure 5). In addition, the observed reduction in MCs levels follows a concentration-dependent pattern. At increasing MO doses of 25 mg L^−1^, 50 mg L^−1^, 75 mg L^−1^, and 100 mg L^−1^, increasing toxins removal of 24.1%, 39.1%, 71.8%, and 88.7% were achieved, respectively. In a related way, the amount of MCs that remains attached to the non-flocculated particulate fraction (dissolved and particulate MCs) was significantly (*p* < 0.05) reduced when increasing the concentration of coagulant applied to the samples (Table 3). The opposite trend was observed for the presence of MCs in the coagulated fraction. In this case, MCs concentration in the residual sludge increased between 3.3- to 8.3- folds (Table 3; Figure 5). All these results could be partially explained considering the above-mentioned decline in the population of *M. aeruginosa* in the aqueous-phase -and its respective increase in the floc when increasing the amount of coagulant added to the samples (Figure 3).

**TABLE 3:** Concentration (µg L^−1^) of MCs soluble in water (dissolved MCs), remaining in the particulate fraction (particulate MCs), and flocculated within the residual sludge (MCs sludge) after *Moringa oleifera* seeds aqueous extract (MO) addition on municipal treated-water samples.

MO (mg L^−1^)	Dissolved MCs (µg L^−1^)	Particulate MCs (µg L^−1^)	MCs in sludge (µg L^−1^)
0	5.5 ± 0.3^a^	28.0 ± 1.6^a^	0.0^e^
25	4.2 ± 0.2^b^	16.0 ± 1.3^b^	4.0 ± 0.8^d^
50	3.4 ± 0.3^c^	10.3 ± 0.8^c^	13.1 ± 1.6^c^
75	1.6 ± 0.2^d^	3.1 ± 0.2^d^	20.0 ± 1.4^b^
100	0.6 ± 0.1^e^	1.0 ± 0.1^d^	33.0 ± 2.5^a^

Data are mean ± S.D. of three independent experiments (*n* = 3). Different lowercase letters in the same column represent significant statistical difference (*p* < 0.05) (*e.g*., “a” and “b” are statistically different from each other).

## 4 Discussion

In the present study, MO showed highly efficient coagulating properties for municipal water treatment. At all four coagulant concentrations evaluated (i.e., 25, 50, 75 and 100 mg L^−1^) the amount of algal and cyanobacterial representatives (Table 1), suspended solids (Figure 4), and soluble MCs content (Table 3) were significantly reduced (*p* < 0.05), compared with the control un-treated water samples. The MO dose of 100 mg L^−1^ achieved a removal reduction of ∼94% in the population of cyanobacteria as well as ∼87% reduction in the population of microalgae present in the municipal treated-water samples. This result agreed with a significant reduction in the Chl *a* content (Table 2), a traditional parameter frequently used as an indicator for evaluating the removal efficiency of microalgal cells from polluted water (Carralero Bon et al., 2021). Different studies have emphasized that *Moringa* seeds yield positively charged aqueous-soluble proteins when mixed with water that can attract the negative charge of the cyanobacterial and microalgal cells promoting floc formation and agglomeration (Gouvea-Barros et al., 2019; Ghernaout et al., 2020; Nhut et al., 2021; Abdul Hamid et al., 2016; Abouzied and Hassan, 2021; Vigneshwaran et al., 2020; Yamaguchi et al., 2021). Moreover, this behaviour may vary depending on differences in algae physiology and morphology, as well as other characteristics such as cell extracellular organic matter composition, surface charge, and algae cell concentration (Li et al., 2018).

Notably, the amount of the harmful cyanobacterium *M. aeruginosa* was 50% reduced with the addition of 25 mg L^−1^ of the natural coagulant (lower tested dose), while a remarkable removal of 90% was achieved at 100 mg L^−1^ MO. These results are promising since *M. aeruginosa* is the most important cause of toxic cyanobacterial blooms affecting humans and animals in inland water systems worldwide (Merel et al., 2013; He et al., 2016). *M. aeruginosa* produce a variety of toxic substances as a result of the cyanobacterial cell energy metabolism including microcystins, micropeptins, linoleic acid, and other secondary metabolites that have been shown to be toxic to aquatic biota affecting the microbial richness and biodiversity (Kaebernick and Neilan, 2001; Sivonen and Hudnell, 2008; Corbel et al., 2014; Rastogi et al., 2014). Accordingly, the observed reduction in the population of *M. aeruginosa* was followed by a decrease in the amount of soluble MCs found in the supernatant of the treated samples after the coagulation process. Consequently, MCs level was concentrated in the coagulated sludge (Table 3) together with the increase in the number of *M. aeruginosa* (Figures 1, 3). Therefore, the later result indicates the need of implementing adequate biosolid treatment procedures for its safe disposal. Finally, it is also noteworthy that some other potentially harmful microalgae, including the toxin-producing cyanoprokaryote *Cylindrospermopsis* was completely removed from the treated-waters even at the lower concentration of 25 mg L^−1^ MO evaluated (Table 1). This result could be related with the algal morphology that may influence the coagulation and sedimentation processes. Larger algal types were reported to be more easily removed than the smaller ones. Accordingly, the filamentous *Cylindrospermopsis* (∼1,500 μm^3^ volume) are larger than the spherical *M. aeruginosa* (∼65 μm^3^). Besides, other cell characteristics such as motility, surface charge, extracellular organic matter composition, and concentration may also influence the coagulant demand (Li et al., 2018).

On the other hand, the aqueous extract of *M. oleifera* seeds here obtained also showed a remarkedly performance for the removal of suspended solids (Figure 4). At the lower dose of MO tested (i.e., 25 mg L^−1^) a turbidity reduction efficiency of ∼70% was achieved. Notably, by doubling the amount of the natural coagulant added to the samples (50 mg L^−1^), the reduction in water turbidity was superior to 90%. In general, these findings agree with previous studies emphasizing the coagulating properties of *M. oleifera* seeds extracts in replacement of traditional coagulants such as permanganate and other metal-based flocculants like iron, copper or aluminum salts (Nhut et al., 2021; Abouzied and Hassan, 2021; Vigneshwaran et al., 2020; Yamaguchi et al., 2021). In addition, these results agreed with the aforementioned reduction in total microalgal biomass from the MO-treated water samples. At this point, it is also important to mention that the sediment load is a frequent problem in water treatment. Synthetic organic and inorganic substances are used at different steps of the clarification process. In most of the cases, these chemicals are required in high doses and after its use most of them generate recalcitrant products that also require further complex treatment. Moreover, many of such compounds are also associated with human health and environmental problems (Bolto and Gregory, 2007). Therefore, the evaluation of cost-effective and environmentally friendly processes for water clarification is still of utmost importance. Regarding this, natural coagulants could be used without the need of any pH adjustment both before and after treatment compared to the traditional metal-containing chemicals used as coagulants/flocculants (Bolto and Gregory, 2007; Abouzied and Hassan, 2021; Vigneshwaran et al., 2020).

Although, natural coagulants are known for their effectiveness in reducing turbidity, little is known about the characteristics and properties of floc formation and the mechanism of microbial removal (Ibrahim et al., 2015; Abdul Hamid et al., 2016; Yamaguchi et al., 2021; Demissie et al., 2021). It has been suggested that the active ingredients that confers coagulating properties to the *Moringa* extracts are cationic peptides that also contribute to microbial elimination and wastewater remediation. Some authors highlighted that *M. oleifera* seeds extracts could form flocs by sorption mechanisms which create bridges and net-like structures between the cationic proteins and the contaminants (Yap et al., 2014; Sun et al., 2020; Ghernaout et al., 2020; Nhut et al., 2021; Abdul Hamid et al., 2016). Therefore, we propose that the cationic peptides present in the MO here obtained neutralize the negative chemical charges at the surface of the algal and cyanobacterial cell walls, and subsequently formed cyanobacterial/algal flocs that finally settle down. Given their high adsorption capacity, MO can also lower both soluble MCs and the cyanotoxins adhered to the particulate fraction in a concentration-dependent manner. These results agree with some previous studies conducted by (Yasmin et al., 2019; Abouzied and Hassan, 2021). However, further studies are still needed to more deeply characterize the cationic peptides present in water-based *M. oleifera* seeds extracts and the kinetics involve in microbial cells removal.

## 5 Conclusion

In the present study, we have demonstrated the usefulness and reliability of *M. oleifera* seeds aqueous extracts to eliminate harmful toxin-producing cyanoprokaryotes in water treatment plants. Our results showed that this locally available natural coagulant significantly reduced the total microalgae content of pre-treated wastewater samples. In addition, suspended solids and soluble cyanotoxins content were also significantly removed to values below the WHO recommended limits for drinking water. Therefore, this natural coagulant still has a bright future in water purification, especially in low-income countries, because of their abundance in nature, low price, environmental-friendly impact, and biodegradability.

## Data Availability

The original contributions presented in the study are included in the article/Supplementary Material, further inquiries can be directed to the corresponding authors.

## Supplementary material

SUPPLEMENTARY FIGURE S1 Representative HPLC-DAD chromatograms obtained from the detection and quantification analyses of MCs **(A)** soluble in MO-treated water samples, **(B)** adhered to the non-flocculated particulate fraction, and **(C)** precipitated within the coagulated sludge. In all cases, the signal corresponding to MCs was detected at a retention time of 1.88 min.
