# Corrigendum: Performance evaluation of *Moringa oleifera* seeds aqueous extract for removing *Microcystis aeruginosa* and microcystins from municipal treated-water

**DOI:** 10.3389/fbioe.2024.1487259

**Published:** 2024-09-17

**Authors:** Haifa A. S. Alhaithloul, Zakaria A. Mohamed, Abdullah A. Saber, Ibtisam Mohammed Alsudays, Mohamed A. Abdein, Mesfer M. Alqahtani, Noha G. AbuSetta, Amr Elkelish, Leonardo Martín Pérez, Fauzeya Mateq Albalwe, Asmaa A. Bakr

**Affiliations:** ^1^ Department of Biology, College of Science, Jouf University, Sakaka, Saudi Arabia; ^2^ Microbiology and Botany Department, Faculty of Science, Sohag University, Sohag, Egypt; ^3^ Botany Department, Faculty of Science, Ain Shams University, Cairo, Egypt; ^4^ Department of Biology, College of Science and Arts, Qassim University, Unaizah, Saudi Arabia; ^5^ Seeds Development Department, El-Nada Misr Scientific Research and Development Projects, Mansoura, Egypt; ^6^ Department of Biological Sciences, Faculty of Science and Humanities, Shaqra University, Shaqraa, Saudi Arabia; ^7^ Microbiology and Botany Department, Faculty of Science, South Valley University, Qena, Egypt; ^8^ Department of Biology, College of Science, Imam Muhammad bin Saud Islamic University (IMSIU), Riyadh, Saudi Arabia; ^9^ Department of Botany and Microbiology, Faculty of Science, Suez Canal University, Ismailia, Egypt; ^10^ Facultad de Química e Ingeniería del Rosario, Pontificia Universidad Católica Argentina (UCA), Rosario, Argentina; ^11^ Laboratory of Environmental and Sanitary Microbiology (MSMLab-UPC), Universitat Politècnica de Catalunya-BarcelonaTech, Terrassa, Spain; ^12^ Department of Biology, Faculty of Science, University of Tabuk, Tabuk, Saudi Arabia

**Keywords:** cyanotoxins, *Microcystis aeruginosa*, *Moringa oleifera*, natural coagulant, water treatment, HPLC-DAD

In the published article, there was an error in the **Funding** statement. The project number 223202 was not included.

The correct **Funding** statement appears below.

