# Supplementary material for: Performance evaluation of Moringa oleifera seeds aqueous extract for removing Microcystis aeruginosa and microcystins from municipal treated-water
Source: Front Bioeng Biotechnol. 2024 Feb 1;11:1329431. doi: 10.3389/fbioe.2023.1329431 (PMC10868579; doi:10.3389/fbioe.2023.1329431)
Supplement: Supplementary file 1 [file Image1.pdf]

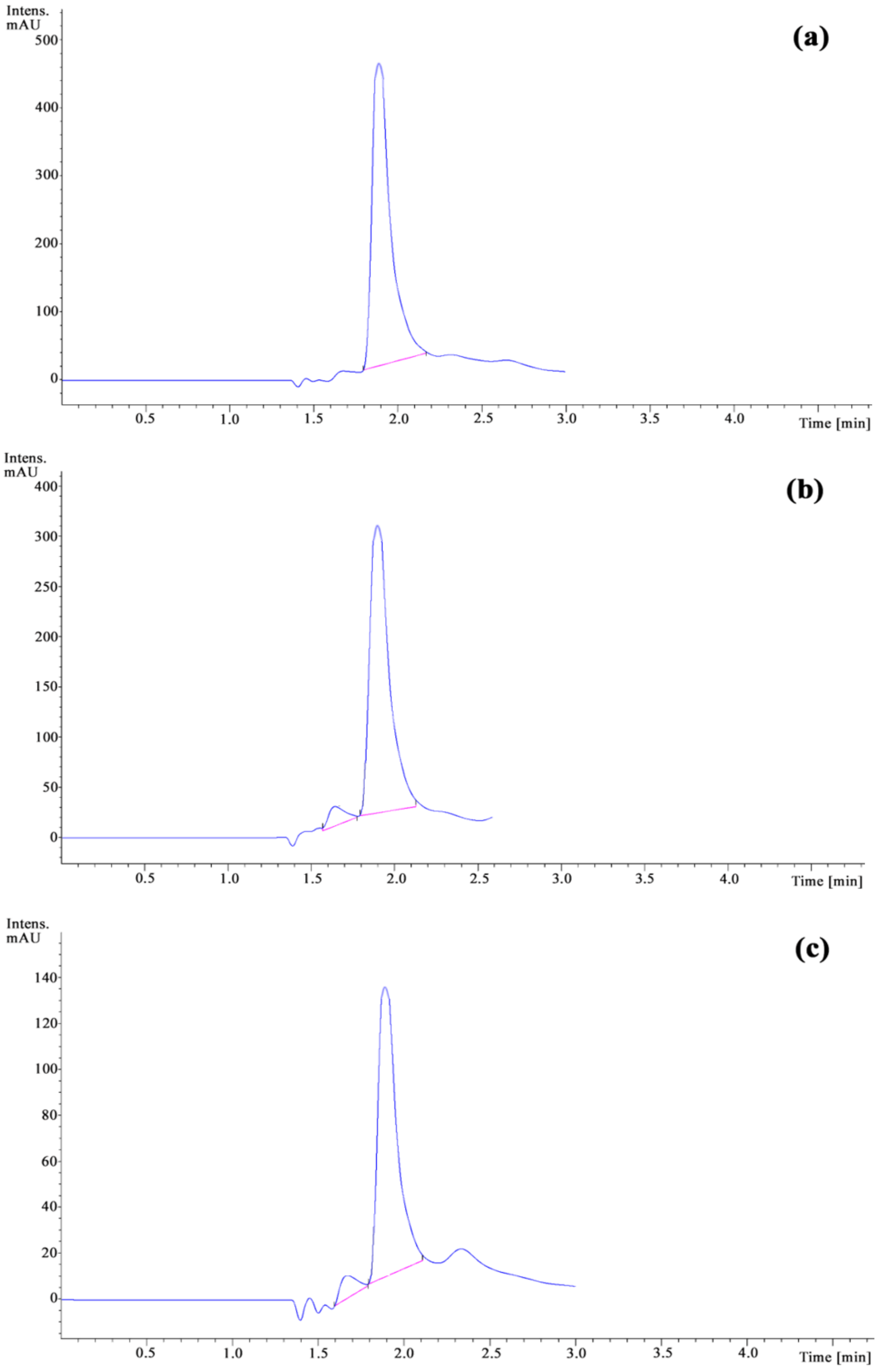

Figure S1. Representative HPLC-DAD chromatograms obtained from the detection and quantification analyses of MCs **(a)** soluble in MO-treated water samples, **(b)** adhered to the non-flocculated particulate fraction, and **(c)** precipitated within the coagulated sludge. In all cases, the signal coresponding to MCs was detected at a retention time of 1.88 min.
